# Bis(4-meth­oxy­pyridin-3-yl)diazene

**DOI:** 10.1107/S1600536812018326

**Published:** 2012-04-28

**Authors:** Steffen Thies, Christian Näther, Rainer Herges

**Affiliations:** a Institut für Organische Chemie, Universität Kiel, Otto-Hahn-Platz 4, 24118 Kiel, Germany; b Institut für Anorganische Chemie, Universität Kiel, Otto-Hahn-Platz 6/7, 24118 Kiel, Germany

## Abstract

The asymmetric unit of the title compound, C_12_H_12_N_4_O_2_, consists of one half-mol­ecule, which is located on a center of inversion. The molecule has a step-like shape; the azo group adopting a *trans* configuration, with the pyridine rings being parallel-displace.

## Related literature

For background to this work, see: Thies *et al.* (2010 ▶, 2011 ▶); Venkataramani *et al.* (2011 ▶).
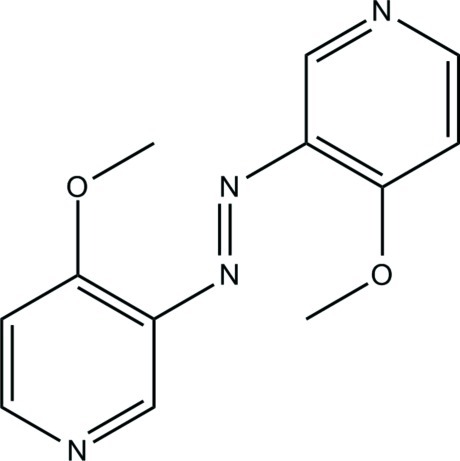


## Experimental

### 

#### Crystal data

C_12_H_12_N_4_O_2_
*M* *_r_* = 244.26
Orthorhombic, 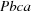

*a* = 13.3976 (8) Å
*b* = 6.2101 (6) Å
*c* = 13.6079 (9) Å
*V* = 1132.18 (15) Å^3^
*Z* = 4
Mo *K*α radiation
μ = 0.10 mm^−1^
*T* = 220 K
0.3 × 0.2 × 0.2 mm

#### Data collection

Stoe IPDS-1 diffractometer
5829 measured reflections
1235 independent reflections
1002 reflections with *I* > 2σ(*I*)
*R*_int_ = 0.045

#### Refinement

*R*[*F* ^2^ > 2σ(*F* ^2^)] = 0.039
*wR*(*F* ^2^) = 0.105
*S* = 1.04
1235 reflections
84 parameters
H-atom parameters constrained
Δρ_max_ = 0.26 e Å^−3^
Δρ_min_ = −0.19 e Å^−3^

### 

Data collection: *X-AREA* (Stoe & Cie, 2008 ▶); cell refinement: *X-AREA*; data reduction: *X-RED32*; program(s) used to solve structure: *SHELXS97* (Sheldrick, 2008 ▶); program(s) used to refine structure: *SHELXL97* (Sheldrick, 2008 ▶); molecular graphics: *XP* in *SHELXTL* (Sheldrick, 2008 ▶); software used to prepare material for publication: *SHELXL97*.

## Supplementary Material

Crystal structure: contains datablock(s) I, global. DOI: 10.1107/S1600536812018326/bt5885sup1.cif

Structure factors: contains datablock(s) I. DOI: 10.1107/S1600536812018326/bt5885Isup2.hkl

Supplementary material file. DOI: 10.1107/S1600536812018326/bt5885Isup3.cml

Additional supplementary materials:  crystallographic information; 3D view; checkCIF report

## Figures and Tables

**Fig. 1.**
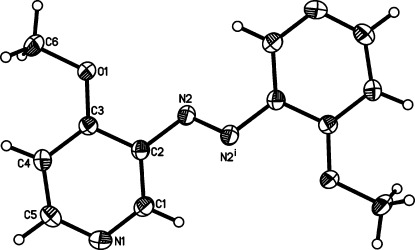
: Crystal structure of the title compound with labeling and displacement ellipsoids drawn at the 50% probability level. Symmetry code: i = -x + 1, -y + 2, -z + 1.

## supplementary crystallographic information

### Comment

We recently reported about a change of the spin state by association/dissociation of photodissociable ligands (PDL's) at square planar Ni(II) porphyrine complexes (Thies *et al.* 2010, Thies *et al.* 2011, Venkataramani *et al.*, 2011). Within this project the title compound was synthesized as potential PDL and its structure was determined by single-crystal X-ray diffraction.

In the crystal structure of the title compound, the azo group is in a *trans* configuration with an torsion angle C2—N2—N2^i^—C2^i^ (i = -*x* + 1, -*y* + 2, -*z* + 1) of 180° due to symmetry. The pyridine rings are not coplanar with the central C—N—N—C unit with torsion angles of 32.9 (2)° for C1—C2—N2—N2^i^ and 151.5 (2)° for C3—C2—N2—N2^i^ (i = -*x* + 1, -*y* + 2, -*z* + 1).

### Experimental

3-Nitro-4-methoxypyridine (1.14 g, 7.41 mmol) was dissolved in ethanol (30 ml) and heated to 80°C. Barium hydroxide (3.50 g, 20.8 mmol) dissolved in 20 ml hot water was added. Zinc powder (6.00 g, 91,7 mmol) was added in small portions within 20 min. The reaction mixture was stirred at 80 °C for 3 h and filtered over celite&reg;. 200 ml dichloromethane were added to the filtrate and air was bubbled through the solution for 2 h. The organic layer was dried over magnesium sulfate and the solvent was removed under reduced pressure. The crude product was dissolved in ethanol and two spoons of activated charcoal were added. After stirring at 80°C for 20 min the mixture was filtered over celite&reg; and the product was crystallized from ethyl acetate. An orange solid (120 mg, 0.49 mmol, 13.2%) was afforded (mp: 218.4 °C). ^1^H-NMR (500 MHz, 300 K, CDCl_3_, TMS)): δ = 8.58 (s, 2H, 2-*H*), 8.55 (d, *J* = 5.8 Hz, 2H, 6-*H*), 7.02 (d, *J* = 5.8 Hz, 2H, 5-*H*), 4.08 (s, 6H, OC*H*_3_) p.p.m.. IR (KBr): ν (cm^-1^) = 2993, 2951, 1562, 1485, 1461, 1442, 1301, 1272, 1014, 817, 762. MS (EI): m/*z* (%) = 244 (100) [*M*]^+^, 136 (66) [*M*-PyrOMe]^+^, 108 (77) [*M*-NNPyrOMe]^+^. MS (CI): m/*z* (%) = 245 (100) [*M*+H]^+^. Anal. Calc.: C_12_H_12_N_4_O_2_ (244.10), ber. C 59.01, H 4.95, N 22.94, gef. C 59.72, H 4.58, N 22.64%.

### Refinement

All H atoms were positioned with idealized geometry (methyl H atoms allowed to rotate but not to tip) and were refined isotropically with *U*_iso_(H) = 1.2 *U*_eq_(C) for aromatic H atoms (1.5 for methyl H atoms) using a riding model.

### Crystal data

**Table:**

C_12_H_12_N_4_O_2_	*D*_x_ = 1.433 Mg m^−^^3^
*M**_r_* = 244.26	Mo *K*α radiation, λ = 0.71073 Å
Orthorhombic, *P**b**c**a*	Cell parameters from 4972 reflections
*a* = 13.3976 (8) Å	θ = 4.7–28.8°
*b* = 6.2101 (6) Å	µ = 0.10 mm^−^^1^
*c* = 13.6079 (9) Å	*T* = 220 K
*V* = 1132.18 (15) Å^3^	Block, colourless
*Z* = 4	0.3 × 0.2 × 0.2 mm
*F*(000) = 512	

### Data collection

**Table:**

Stoe IPDS-1 diffractometer	1002 reflections with *I* > 2σ(*I*)
Radiation source: fine-focus sealed tube	*R*_int_ = 0.045
Graphite monochromator	θ_max_ = 27.1°, θ_min_ = 3.0°
Phi scans	*h* = −17→12
5829 measured reflections	*k* = −7→7
1235 independent reflections	*l* = −17→17

### Refinement

**Table:**

Refinement on *F*^2^	Secondary atom site location: difference Fourier map
Least-squares matrix: full	Hydrogen site location: inferred from neighbouring sites
*R*[*F*^2^ > 2σ(*F*^2^)] = 0.039	H-atom parameters constrained
*wR*(*F*^2^) = 0.105	*w* = 1/[σ^2^(*F*_o_^2^) + (0.0625*P*)^2^ + 0.204*P*] where *P* = (*F*_o_^2^ + 2*F*_c_^2^)/3
*S* = 1.04	(Δ/σ)_max_ < 0.001
1235 reflections	Δρ_max_ = 0.26 e Å^−^^3^
84 parameters	Δρ_min_ = −0.19 e Å^−^^3^
0 restraints	Extinction correction: *SHELXL97* (Sheldrick, 2008), Fc^*^=kFc[1+0.001xFc^2^λ^3^/sin(2θ)]^-1/4^
Primary atom site location: structure-invariant direct methods	Extinction coefficient: 0.030 (8)

### Special details

**Table:**

Geometry. All e.s.d.'s (except the e.s.d. in the dihedral angle between two l.s. planes) are estimated using the full covariance matrix. The cell e.s.d.'s are taken into account individually in the estimation of e.s.d.'s in distances, angles and torsion angles; correlations between e.s.d.'s in cell parameters are only used when they are defined by crystal symmetry. An approximate (isotropic) treatment of cell e.s.d.'s is used for estimating e.s.d.'s involving l.s. planes.
Refinement. Refinement of *F*^2^ against ALL reflections. The weighted *R*-factor *wR* and goodness of fit *S* are based on *F*^2^, conventional *R*-factors *R* are based on *F*, with *F* set to zero for negative *F*^2^. The threshold expression of *F*^2^ > σ(*F*^2^) is used only for calculating *R*-factors(gt) *etc*. and is not relevant to the choice of reflections for refinement. *R*-factors based on *F*^2^ are statistically about twice as large as those based on *F*, and *R*- factors based on ALL data will be even larger.

### Fractional atomic coordinates and isotropic or equivalent isotropic displacement parameters (Å^2^)

**Table:**

	*x*	*y*	*z*	*U*_iso_*/*U*_eq_	
N1	0.66974 (9)	0.6358 (2)	0.34371 (9)	0.0314 (3)	
C1	0.62249 (10)	0.7907 (2)	0.39360 (9)	0.0263 (3)	
H1	0.6591	0.9134	0.4119	0.032*	
C2	0.52211 (9)	0.7811 (2)	0.42010 (8)	0.0211 (3)	
C3	0.46546 (10)	0.60269 (19)	0.38987 (8)	0.0205 (3)	
C4	0.51423 (10)	0.4414 (2)	0.33786 (9)	0.0250 (3)	
H4	0.4794	0.3190	0.3161	0.030*	
C5	0.61506 (11)	0.4644 (2)	0.31870 (10)	0.0301 (3)	
H5	0.6475	0.3513	0.2857	0.036*	
N2	0.47208 (8)	0.94965 (17)	0.47068 (7)	0.0228 (3)	
O1	0.36924 (7)	0.60036 (15)	0.41627 (7)	0.0277 (3)	
C6	0.30947 (11)	0.4230 (2)	0.38338 (11)	0.0321 (4)	
H6A	0.3032	0.4284	0.3124	0.048*	
H6B	0.2438	0.4320	0.4130	0.048*	
H6C	0.3410	0.2888	0.4024	0.048*	

### Atomic displacement parameters (Å^2^)

**Table:**

	*U*^11^	*U*^22^	*U*^33^	*U*^12^	*U*^13^	*U*^23^
N1	0.0230 (6)	0.0348 (6)	0.0364 (7)	−0.0008 (5)	0.0067 (5)	−0.0042 (5)
C1	0.0237 (7)	0.0271 (7)	0.0280 (6)	−0.0044 (5)	0.0010 (5)	−0.0020 (5)
C2	0.0227 (6)	0.0202 (6)	0.0203 (5)	0.0000 (5)	−0.0014 (4)	−0.0006 (4)
C3	0.0200 (6)	0.0208 (6)	0.0205 (6)	0.0011 (5)	−0.0014 (4)	0.0011 (4)
C4	0.0273 (7)	0.0216 (6)	0.0261 (6)	−0.0009 (5)	−0.0004 (5)	−0.0045 (5)
C5	0.0292 (7)	0.0283 (7)	0.0329 (7)	0.0039 (6)	0.0052 (5)	−0.0058 (5)
N2	0.0231 (5)	0.0202 (5)	0.0251 (5)	−0.0017 (4)	−0.0012 (4)	−0.0022 (4)
O1	0.0193 (5)	0.0241 (5)	0.0396 (6)	−0.0030 (4)	0.0020 (4)	−0.0086 (4)
C6	0.0230 (7)	0.0288 (7)	0.0444 (8)	−0.0068 (6)	−0.0037 (6)	−0.0068 (6)

### Geometric parameters (Å, º)

**Table:**

N1—C5	1.3360 (19)	C4—C5	1.3832 (19)
N1—C1	1.3369 (18)	C4—H4	0.9400
C1—C2	1.3936 (18)	C5—H5	0.9400
C1—H1	0.9400	N2—N2^i^	1.260 (2)
C2—C3	1.4044 (17)	O1—C6	1.4333 (16)
C2—N2	1.4209 (16)	C6—H6A	0.9700
C3—O1	1.3384 (16)	C6—H6B	0.9700
C3—C4	1.3897 (17)	C6—H6C	0.9700
			
C5—N1—C1	116.29 (12)	C3—C4—H4	120.6
N1—C1—C2	123.89 (12)	N1—C5—C4	124.74 (13)
N1—C1—H1	118.1	N1—C5—H5	117.6
C2—C1—H1	118.1	C4—C5—H5	117.6
C1—C2—C3	118.65 (11)	N2^i^—N2—C2	113.10 (14)
C1—C2—N2	123.30 (11)	C3—O1—C6	117.56 (10)
C3—C2—N2	117.91 (11)	O1—C6—H6A	109.5
O1—C3—C4	125.58 (11)	O1—C6—H6B	109.5
O1—C3—C2	116.77 (11)	H6A—C6—H6B	109.5
C4—C3—C2	117.62 (12)	O1—C6—H6C	109.5
C5—C4—C3	118.73 (12)	H6A—C6—H6C	109.5
C5—C4—H4	120.6	H6B—C6—H6C	109.5

Symmetry code: (i) −*x*+1, −*y*+2, −*z*+1.
